# The effectiveness of gender-neutral HPV vaccination programmes in preventing HPV-associated oral cancers: a systematic review

**DOI:** 10.1186/s12885-026-15979-3

**Published:** 2026-04-16

**Authors:** Lamia M. Douyat, Angela Spencer

**Affiliations:** Division of Population Health, Health Services Research and Primary Care, School of Health Sciences, University of Manchester, Ellen Wilkinson Building, Oxford Road, Manchester, M13 9PR UK

**Keywords:** Human Papillomavirus, Oral cancer, Oropharyngeal cancer, Cancer prevention, Vaccination programs, Gender-neutral vaccination, Vaccine Type HPV

## Abstract

**Background:**

Human papillomavirus infections are a significant risk factor for the development of oral cancers. While HPV vaccines have proven effective in preventing cervical cancers, their impact on oral cancers remains insufficiently examined. This systematic review assesses the effectiveness of gender-neutral HPV vaccination in reducing oral HPV rates, a key precursor to HPV-associated oral cancers, focusing exclusively on studies including gender-neutral samples.

**Methods:**

Following PRISMA 2020 guidelines, a comprehensive search strategy was implemented, covering multiple databases, including MEDLINE and Embase, as well as grey literature and ongoing trials sources via WHO ICTRP, EUCTR and ClinicalTrials.gov. Inclusion criteria encompassed studies published between 2009 and 2025 with gender-neutral samples, oral HPV prevalence data, and vaccination status information. Quality of the included studies was assessed using the JBI Critical Appraisal Checklists.

**Results:**

Nine observational studies were included, with sample sizes ranging from 394 to 9,437 participants and age ranges spanning from 18 to 70 years across multiple countries, including the United States, Australia, and Italy. Six studies reported lower prevalence of oral vaccine-type HPV (VT-HPV) among vaccinated individuals, with relative reductions ranging from 57.5% to 93.2% and an overall mean reduction of 78.5%. Lower prevalence was observed even after a single vaccine dose. In sex-stratified analyses, females showed a mean reduction of 86.8%, while males showed a mean reduction of 69.1%, with two studies reporting 100% protection among vaccinated males. Heterogeneity in outcome definitions, reporting formats, and sample overlap precluded meta-analysis.

**Conclusions:**

This is the first systematic review focusing exclusively on gender-neutral samples. The findings suggest that gender-neutral HPV vaccination programmes, which are associated with lower oral prevalence of vaccine-type HPV, may contribute to reducing the burden of HPV-associated oral cancers. Including males in national immunisation strategies is essential to promote equity and maximize public health impact, particularly given the higher burden of HPV-associated oropharyngeal cancers in men. Further research is required to better understand this effect, including the influence of dose number and the potential role of sex in vaccine response.

**Registration:**

PROSPERO (CRD420251144148).

**Supplementary Information:**

The online version contains supplementary material available at 10.1186/s12885-026-15979-3.

## Background

Oral Cancers (OCs) are malignancies of the oral cavity, lips, and oropharynx, as defined by World Health Organization (WHO) and World Dental Federation (FDI) [1–2]. The most common histological type of OCs is squamous cell carcinoma [3].

In 2022, oral cavity and lip cancers accounted for 389,485 new cases and 188,230 cancer-related deaths globally, representing approximately 2.0% of all new cancer diagnoses and 1.9% of all cancer-related mortality [4]. In this same year, Oropharyngeal Cancers (OPCs) constituted of 106,316 new cases and 52,268 deaths, or 0.5% of the global incidence and 0.5% of the mortality rate [4]. These statistics highlight the significant impact of OCs on public health and the urgent need for effective prevention, early detection, and treatment strategies. OCs exhibit marked sex disparities, with males being approximately three times more likely than females to develop OCs, particularly OPCs [5, 6]. In 2022, males accounted for 268,759 new oral cavity and lip cancer cases and 86,269 oropharyngeal cases, whereas females had 120,726 and 20,047 cases, respectively [4].

Signs and symptoms of OCs include persistent ulcers, lumps, and white or red patches on the gingiva, tongue, lips or lining of the mouth, with difficulty swallowing or speaking [7, 8]. If not diagnosed and treated early, OCs can be aggressive and life-threatening as they may metastasize [7, 8]. Treatment options involve surgery, radiation, and chemotherapy, depending on stage and extent [9]. The development of OCs is strongly linked to risk factors such as tobacco and alcohol use [8]. Other risk factors include sun exposure and poor diet [8]. Although the incidence of oral cancers associated with these factors has declined, human papillomavirus (HPV) has emerged as a significant etiological factor, particularly in OPCs, where it is now considered the leading cause [10, 11]. HPV infection accounts for approximately 31% of OPCs and 2.1% of oral cavity cancers [12]. HPV16 and, to a lesser extent, HPV18 play significant role in the onset of OCs [13]. Evidence suggests that more than 90% of virus-driven head and neck squamous cell carcinomas are attributable to HPV16 [12].

HPV is a sexually transmitted virus that causes genital and oral infections that typically resolve spontaneously, but persistent infections increase the risk of developing cancers such as cervical, anal, vaginal, penile, vulvar, and oral cancers [14]. According to 2018 estimates, HPV was responsible for nearly 690,000 new cancer cases globally, accounting for an age-standardised incidence rate (ASIR) of 8.0 cases per 100,000 person-years [15]. Cervical cancer represents 80% of this HPV attributable cancer burden, while OCs contribute around 7% [15]. Recent evidence suggests a rising incidence of HPV-associated OPCs among younger adults, with an annual percentage change of 0.8% (*P* < 0.001) between 1973 and 2004 [16].

HPV is highly prevalent worldwide; with over 80% of men and women estimated to acquire at least one form of HPV infection during their lifetime [17]. The prevalence of high-risk oral HPV infection is higher in men (6.8%) than in women (1.2%) [18].

The first HPV vaccine, Gardasil, was licensed in 2006 to provide protection against low-risk HPV 6 and 11 and high-risk HPV 16 and 18. In 2007, Cervarix was introduced, targeting HPV 16 and 18. Gardasil 9 was licensed in 2014 and expanded coverage to nine HPV types: 6, 11, 16, 18, 31, 33, 45, 52, and 58 [19]. Two additional HPV vaccines have received WHO prequalification in recent years. Cecolin, a bivalent vaccine, was prequalified in 2021 and approved for single-dose schedules in October 2024, although this constitutes off-label use [20, 21]. Walrinvax, which is also a bivalent vaccine, became the fifth WHO- prequalified HPV vaccine in 2024 [20, 21]. There are also nationally licensed vaccines that have been developed and used regionally. These are not yet WHO-prequalified but play important roles in national immunisation strategies, such as Cervavac in India [22]. These additions enhance the diversity and availability of HPV vaccine options for immunisation programmes worldwide.

HPV vaccines were initially developed for the prevention of cervical cancer; however, they target high-risk HPV types, particularly HPV 16 and 18, which are implicated in oral and oropharyngeal carcinogenesis [13, 19].

National HPV vaccination programs were implemented between 2006 and 2008, offering the vaccine to adolescent girls as part of cervical cancer prevention, with Australia, the United Kingdom, the United States, and Canada being among the first [23]. HPV vaccines have proven effective in preventing cervical cancers. No cases of invasive cervical cancer were observed in women vaccinated at age 12–13 years compared with unvaccinated cohorts in a Scottish population-based study [24]. In a nationwide Swedish cohort study including 1.7 million women, HPV vaccination before the age of 17 years was associated with an 88% lower cervical cancer incidence compared with unvaccinated women [25]. The aim of enhancing cervical cancer prevention by providing indirect protection through herd immunity, along with the rise in HPV-associated cancers among males, has driven the expansion of HPV vaccination programmes, with Australia launching the first gender-neutral programme in 2013 [26, 27]. Following WHO’s 2017 recommendations, 80 countries now have gender-neutral HPV vaccination programmes [28].

This systematic review aims to evaluate the effectiveness of gender-neutral HPV vaccination programmes in preventing HPV-associated OCs. By analysing data on the prevalence of detectable oral HPV based on vaccination status across selected studies, this review will assess the overall impact of HPV vaccines and explore differences in vaccine effectiveness between genders.

A previous systematic review explored the effectiveness of HPV vaccination in men, with the broader aim of informing pangender vaccination strategies [29]. The authors concluded that HPV vaccination was associated with reduced oral HPV positivity, which was interpreted as suggestive of a potential reduction in the risk of developing HPV-associated OPC. While the review emphasised the importance of implementing gender-neutral HPV vaccination strategies, it also acknowledged that long-term effects on OPC incidence remain uncertain due to the relatively recent inclusion of males in vaccination programmes and the long latency of disease development [29]. The analysis also combined male-only studies with those including both sexes, and its scope was restricted to evidence available up to 2021 [29].

In contrast, the present review applies more stringent inclusion criteria by focusing exclusively on gender-neutral samples and excluding single-gender studies, ensuring that findings reflect population-level gender-neutral vaccination outcomes. Moreover, by extending the search to February 2025, this review captures newer studies conducted after the expansion of male HPV vaccination into national immunisation programmes, such as in the United Kingdom in 2019 and the Netherlands in 2022 [30, 31].

Prior to this policy shift, HPV vaccination for males was available, but it was a matter of individual choice. Its integration into national immunisation programmes can exponentially increase vaccine’s uptake among males, as has previously been demonstrated for females [32, 33]. Consequently, newer studies are likely to provide more robust data on vaccinated male populations, allowing for a clearer assessment of gender-neutral vaccination effectiveness.

While other systematic reviews with similar research question have included studies reporting the incidence of OCs or OPCs as an outcome, this review narrowed its focus to studies reporting the prevalence and incidence of oral HPV infections. The rationale behind this decision lies in the multifactorial nature of OCs and OPCs, which can be caused by various causes aside from HPV infections, such as tobacco and alcohol consumption [8]. This approach aims to reduce potential bias arising from the inclusion of cases unrelated to HPV. Additionally, investigating the vaccine’s effectiveness in reducing the incidence of OCs or OPCs is a complex task requiring prolonged follow-up periods, given that the development of HPV-associated OCs typically occurs years after initial infection and they most commonly affect older ages (> 50) [29, 34]. Because premalignant lesions in the oral cavity are difficult to identify, oral HPV infection, particularly with high-risk types, provides a more immediate and specific marker for evaluating the impact of HPV vaccination on HPV-associated OCs [12, 13].

## Methods

This systematic review adheres to the Preferred Reporting Items for Systematic Reviews and Meta-Analyses (PRISMA) 2020 guidelines [35]. The protocol was registered in PROSPERO (ID: CRD420251144148).

### Search strategy

MEDLINE and Embase via Ovid were systematically searched to identify relevant studies. Grey literature and trial registries were explored such as the World Health Organization’s International Clinical Trials Registry Platform (WHO ICTRP), European Union Clinical Trials Register (EUCTR), and ClinicalTrials.gov. This aimed to broaden the scope of research findings beyond conventional journal publications, thereby maximizing the coverage. Each of these sources was searched in February 2025, including backward citation screening within included studies. The search strategy was designed to capture both MeSH terms and free-text keywords related to HPV vaccination and oral/oropharyngeal outcomes, including terms for HPV vaccines (e.g., “Papillomavirus vaccine”, “bivalent”, “quadrivalent”, “9-valent”, “Cervarix”, “Gardasil”) and outcome terms (e.g., “HPV infections”, “neoplasms”, “cancer”, “malignancy”) combined with “mouth” and “oropharynx”. Boolean operators (AND, OR) and truncation (*) were strategically employed to structure the queries where needed. A year range filter (2009–2025) was applied to limit results to studies conducted after the Food and Drug Administration (FDA)’s approval of the quadrivalent HPV vaccine for males in 2009, ensuring relevance to gender-neutral vaccination programmes [36]. The detailed search strategies for each database are provided in Additional File 1.

### Eligibility criteria

Eligibility criteria were defined using the Patient, Intervention, Comparison, Outcome, and Study design (PICOS) framework. Studies published in English with full-text availability between January 2009 and 07 February 2025 were considered eligible if they assessed the impact of HPV vaccination on oral HPV infection rates in gender-neutral populations. Eligible studies included males and females of any age with documented HPV vaccination status (bivalent, quadrivalent, or 9-valent vaccines) and reported the prevalence or incidence of oral or oropharyngeal HPV infection. Randomised controlled trials and observational studies, including cross-sectional, cohort, case–control, and registry-based designs, were included if they compared outcomes between vaccinated and unvaccinated individuals. Grey literature was considered when full-text data were available. No restrictions were placed on study setting or geographic location.

Studies were excluded if they involved single-gender populations, did not assess oral or oropharyngeal HPV infection, or did not directly link vaccination status to oral HPV outcomes. Additional exclusions included studies reporting cancer outcomes without infection data, immunogenicity outcomes only, or modelling-based estimates rather than empirical data.

Qualitative research, animal or in vitro studies, and publication types without primary data, such as systematic reviews, editorials, opinion pieces, or conference abstracts, were also excluded. Studies exclusively involving high-risk populations, such as HIV patients, men who have sex with men (MSM), sex workers, or people who use drugs, were also excluded to improve generalisability to populations targeted by national gender-neutral HPV vaccination programmes. Studies were grouped according to outcome measure (prevalence or incidence of oral HPV infection).

### Study selection

Mendeley Desktop software was used to manage records and remove duplicates. Titles and abstracts were screened by one reviewer (LD), with a random 10% independently screened by a second reviewer (AS) to ensure consistency. Subsequently, both reviewers independently assessed the full texts of potentially eligible studies. Discrepancies were resolved through discussion during regular meetings. Grey literature results were manually screened by both reviewers using an Excel spreadsheet due to the limited number of records retrieved. Reference lists of included studies were screened manually by one reviewer to identify any additional relevant articles.

### Data extraction and synthesis

Data from the included studies were extracted into four tables using Microsoft Word. One reviewer (LD) extracted the data, which were cross-checked by a second reviewer (AS), with discrepancies resolved through discussion. When data were unclear or missing, no assumptions were made and values were recorded as NR (not reported). As meta-analysis was not feasible, vaccine effects were summarised by calculating the relative reduction in oral HPV prevalence between vaccinated and unvaccinated groups. This was calculated as:   

$$\:\frac{\mathrm{P}\mathrm{r}\mathrm{e}\mathrm{v}\mathrm{a}\mathrm{l}\mathrm{e}\mathrm{n}\mathrm{c}\mathrm{e}\:\left(\mathrm{u}\mathrm{n}\mathrm{v}\mathrm{a}\mathrm{c}\mathrm{c}\mathrm{i}\mathrm{n}\mathrm{a}\mathrm{t}\mathrm{e}\mathrm{d}\right)-\:\mathrm{P}\mathrm{r}\mathrm{e}\mathrm{v}\mathrm{a}\mathrm{l}\mathrm{e}\mathrm{n}\mathrm{c}\mathrm{e}\:\left(\mathrm{v}\mathrm{a}\mathrm{c}\mathrm{c}\mathrm{i}\mathrm{n}\mathrm{a}\mathrm{t}\mathrm{e}\mathrm{d}\right)}{\mathrm{P}\mathrm{r}\mathrm{e}\mathrm{v}\mathrm{a}\mathrm{l}\mathrm{e}\mathrm{n}\mathrm{c}\mathrm{e}\:\left(\mathrm{u}\mathrm{n}\mathrm{v}\mathrm{a}\mathrm{c}\mathrm{c}\mathrm{i}\mathrm{n}\mathrm{a}\mathrm{t}\mathrm{e}\mathrm{d}\right)}\:\times\:100\:$$

This measure corresponds to the percent attributable risk in the exposed and is equivalent to standard expressions of vaccine efficacy or effectiveness [37]. Means and medians were calculated where applicable. When studies reported oral vaccine-type HPV (VT-HPV) prevalence by dose, subgroup estimates were averaged to produce a single summary estimate per study to avoid over-representation.

### Quality assessment

Risk of bias was assessed using the Joanna Briggs Institute (JBI) Critical Appraisal Tools 2017 [38, 39]. Studies meeting a minimum threshold of ≥ 6 out of 8 on the cross-sectional checklist or ≥ 9 out of 11 on the cohort checklist were considered to have a low risk of bias and were included. Figures summarising the JBI risk of bias assessments were created using Microsoft Word and presented in the results.

## Results

### Search results

**Fig. 1:**
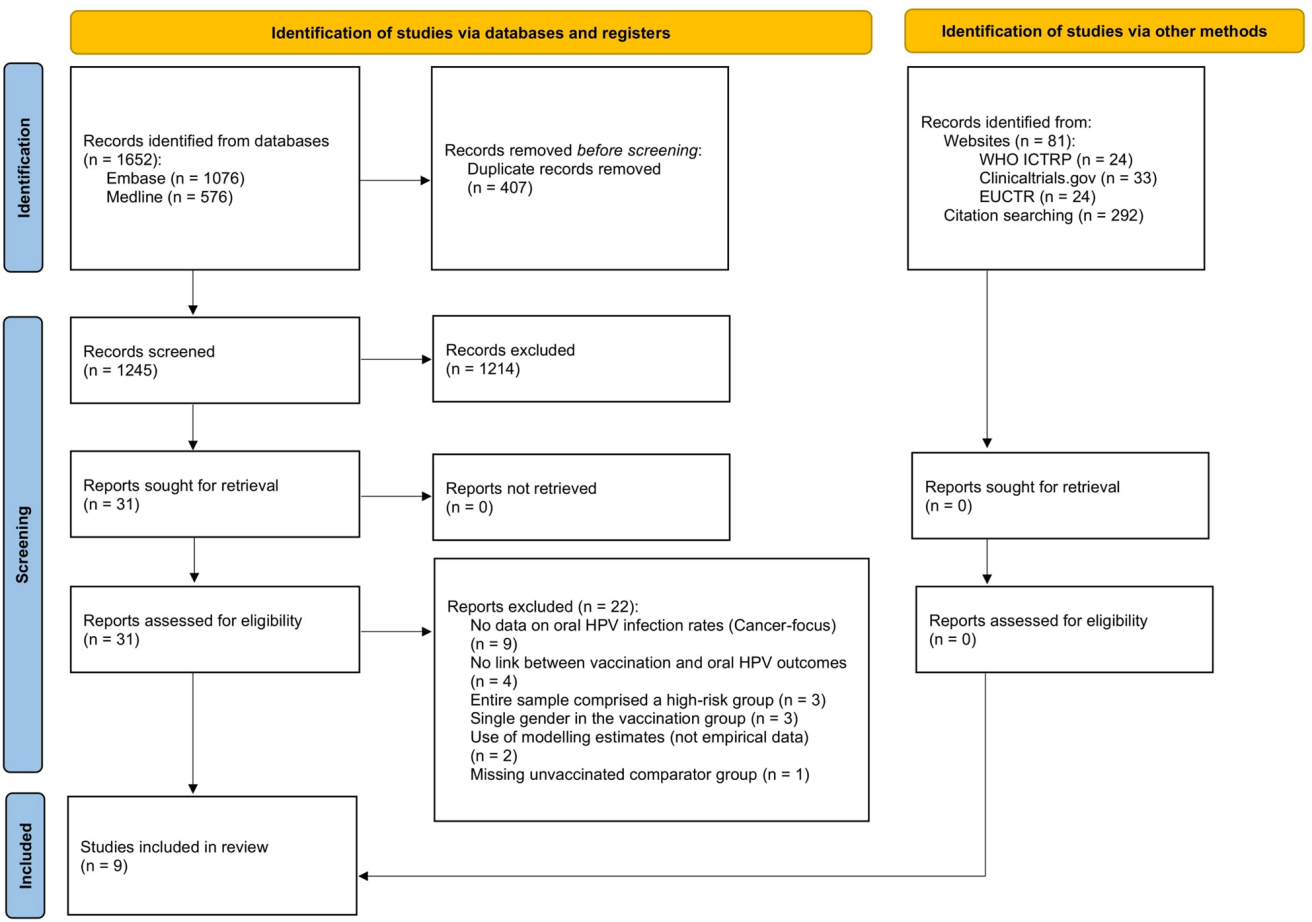
PRISMA flow diagram illustrating the study selection process. Records were identified through database searching (MEDLINE and Embase via Ovid), trial registries, and citation searching. After duplicate removal and screening, 31 reports were assessed for eligibility, of which 9 were included. Reasons for exclusion are found in the figure and Additional file 2

A literature search conducted on 7 February 2025 in MEDLINE and Embase via Ovid identified 1,652 records (Embase: 1,076; MEDLINE: 576). After removal of 402 duplicates in Mendeley and a further 5 duplicates during title and abstract screening, 1,245 records remained for title and abstract screening. Subsequently, 31 studies were selected for full-text review, of which 9 met the inclusion criteria [40–48]. No reports were excluded due to retrieval issues, and automation tools were not used in the screening process. Searches of trial registries identified 81 records across ClinicalTrials.gov (33), WHO ICTRP (24), and EUCTR (24), none of which met the inclusion criteria. Reference list screening of included studies yielded no additional eligible records. The study selection process is summarised in the PRISMA flow diagram (Fig. 1), and reasons for full-text exclusions are provided in Additional file 2.

### Study characteristics

Among the nine included studies, seven were cross-sectional [40–44, 47, 48], one represented the baseline phase of a longitudinal cohort study [45], and one was a longitudinal cohort study [46]. Sample sizes varied significantly, ranging from 317 to 9437 participants. Five studies focused on younger adults, with minimum ages of 18 and maximum ages ranging from 24 to 36 years, while four studies included broader adult age ranges extending from 18 to 50–70 years. Consequently, in this review, the lower age limit was consistently 18 years. However, the upper age limit could not be precisely established, as two studies reported the range as 18–50 + without specifying a maximum age [45, 46]. Seven studies were conducted in the United States (U.S.) [40–46], five of which utilized data from the National Health and Nutrition Examination Survey (NHANES) datasets to assess vaccination status, demographics, and oral HPV prevalence [40–44]. The remaining studies were conducted in Australia and Italy [47, 48]. The characteristics of the included studies are summarized in Table 1. Additional details on sample sizes, gender distribution, and vaccination status are provided in Table 2.

**Table 1:** Characteristics of the included studies

Authors and publication year	Hirth et al. 2017 [40]	Chaturvedi et al. 2018 [41]	Brouwer et al. 2019 [42]	Abel et al. 2021 [43]	Berenson et al. 2022 [44]	Brouwer et al. 2022a [45]	Brouwer et al. 2022b [46]	De Souza et al. 2023 [47]	Napolitano et al. 2024 [48]
Study design	Cross-sectional	Cross-sectional	Cross-sectional	Cross-sectional	Cross-sectional	Longitudinal cohort (Baseline phase)	Longitudinal cohort	Cross-sectional	Cross-sectional
Sample size (N)	*N* = 3040	*N* = 2627	*N* = 1802 (Oral HPV subgroup; not the full cohort)	*N* = 5798	*N* = 9437	*N* = 394 (338 had valid oral HPV tests)	*N* = 394 (317 are included in the oral HPV analysis)	*N* = 911	*N* = 1182 (Provided valid saliva samples)
Age group	18–30	18–33	18–24*	18–36	18–59	18–50+	18–50+	18–70	18–30
Location	USA	USA	USA	USA	USA	USA	USA	Australia	Italy
Data sources / Year	Based on data from NHANES conducted between 2009 and 2014.	Based on data from NHANES conducted between 2011 and 2014.	Based on data from NHANES conducted between 2009 and 2014.	Based on data from NHANES conducted between 2009 and 2016.	Based on data from NHANES conducted between 2011 and 2016.	Questionnaires. 2018–2020.	Questionnaires and follow-ups, 2015–2017	Questionnaires and the Australian Immunisation Register (AIR). 2020–2021.	Questionnaires and self-samples, 2022–2023
Research question	Compare vaccine-type oral HPV prevalence in vaccinated vs. unvaccinated young adults.	Investigate the impact of HPV vaccination on the prevalence of oral HPV infections among young adults.	Estimate HPV prevalence (oral/genital) and genotype concordance across sites, stratified by sex and vaccination status.	Investigate differences in oral HPV prevalence by number of vaccine doses received.	Assess oral HPV prevalence across vaccinated vs. unvaccinated individuals by sex and race.	Assess oral and cervico-genital HPV prevalence by vaccination status and other factors in the MHOC cohort.	Estimate incidence and clearance of oral and cervico-genital HPV over time.	Compare oral HPV prevalence by vaccination status in an Australian sample.	Assess genotype-specific oral and genital HPV prevalence by vaccination status.
Outcome of interest	Prevalence of oral HPV types, focusing on vaccine types (6, 11, 16, 18).	Prevalence of oral HPV types, focusing on vaccine types (6, 11, 16, 18).	Prevalence and concordance of oral and genital HPV infections.	Prevalence of oral HPV types, focusing on vaccine types (6, 11, 16, 18).	Prevalence of different oral HPV types.	Prevalence of oral and cervico-genital HPV types.	Incidence and clearance of oral and cervico-genital HPV types.	Prevalence of different oral HPV types.	Prevalence of oral and genital HPV types.
Key findings	Reduction in oral HPV (4-valent types) prevalence in the vaccinated group.	Significant reduction in oral HPV (4-valent types) prevalence in the vaccinated group.	Strong protective effect of the vaccine against oral HPV in vaccinated men and women. Genital HPV infections show no concordance with oral infections, whereas oral infections show higher concordance with genital sites.	Reduction in oral HPV prevalence in the vaccinated group. No difference between 1 vs. 2–3 doses. Higher prevalence in smokers, early oral sex, and > 2 partners.	Reduction in oral HPV prevalence in the vaccinated group. Higher prevalence among males and the ethnic group: Black.	No significant overall difference by vaccination status, but lower HPV prevalence observed among vaccinated participants within specific age groups. Higher oral and cervico-genital HPV with more recent sexual partners.	No significant reduction in oral HPV incidence by vaccination. Significant reduction in cervico-genital HPV incidence by vaccination. Oral HPV infections were mostly transient; cleared rapidly.	Significant reduction in oral HPV (9-valent types) prevalence in the vaccinated group. Oral HPV infection is strongly associated with sexual activity.	Vaccination is associated with lower oral HPV prevalence. Behaviour is strongly linked.

*The full study age range is 18–69 years, but numerical data are reported only for the 18–24 age group

**Table 2:** Sample sizes, gender distribution, and vaccination data for the included studies

	Sample Size *N* (% of total sample) *			Vaccinated Participants *N* (% of total sample) *			Unvaccinated Participants *N* (% of total sample) *		
	Total	Males	Females	Total	Males	Females	Total	Males	Females
Hirth et al. 2017 [40]	3040	1154 (39.3%)	1886 (60.7%)	668 (22%)	(16.1%)	(83.9%)	2372	(45.8%)	(54.2%)
Chaturvedi et al. 2018 [41]	2627	1328	1299	496	102	394	2131	1226	905
Brouwer et al. 2019 [42]	1802	693	1109	520	90	430	1282	603	679
Abel et al. 2021 [43]**	5798	NR^†^	(58.6%)	997			4801		
Berenson et al. 2022 [44]	9437	4550	4887	939 (9.2%)	216	723	8498 (90.8%)	4334	4164
Brouwer et al. 2022a [45]**	338^‡^	110	228	159^‡^			159^‡^		
Brouwer et al. 2022b [46]**	317^‡^	101 (32%)	216 (68%)	152 (48%)^‡^			142 (45%)^‡^		
De Souza et al. 2023 [47]**	901	227 (24.9%)	678 (74.4%)	230 (25.5%)			671 (74.5%)		
		Non-binary = 6 (0.7%)							

NR Not reported

* Percentages (%) shown in brackets are reported only when explicitly stated in the original studies. No values were calculated by the reviewers

** Sex-specific vaccination data were not reported in the study

^†^ Although the proportion of males can be inferred from the reported percentage of females, values are marked as not reported (NR) to maintain consistency with the review’s policy of not calculating or inferring percentages not explicitly provided in the original publications

^‡^ Vaccination status was not reported for all participants; therefore, the sum of vaccinated and unvaccinated individuals is less than the total sample size

All included studies enrolled both male and female participants, and one study additionally reported a small number of non-binary participants [47]. One study, which included approximately two-thirds female participants, was eligible for inclusion in the review; however, sex-specific participant breakdowns for the saliva samples used in oral HPV testing were not reported [48]. As a result, the study could not be presented in Table 2 but is included in other tables.

Sample sizes ranged from 317 to 9,437 participants. Aggregate totals were not calculated to avoid double-counting, as several studies were derived from the same data sources. Five studies [40–44] were based on overlapping NHANES datasets collected between 2009 and 2016, and two publications [45, 46] reported on the same cohort at baseline and follow-up. The proportion of male participants varied between 25.2% and 50.6% across studies that reported sex distribution. Vaccination uptake ranged from 9.2% to 48.9%. While all studies provided data for vaccinated and unvaccinated groups, five studies did not report vaccination status disaggregated by sex [43, 45–48]. Data available in the original reports are presented in Table 2.

### Risk of bias assessment

Risk of bias was assessed for all nine included studies using the appropriate JBI Critical Appraisal Checklists: the analytical cross-sectional checklist and the cohort studies checklist [38, 39]. Seven studies were cross-sectional, one was a longitudinal cohort study, and one represented the baseline phase of a longitudinal cohort study [45]. As the latter collected data at a single point in time; providing a glimpse into the study population, its methodology aligned more closely with a cross-sectional approach [49]. Therefore, eight studies were assessed using the cross-sectional checklist and one using the cohort checklist. Overall, all studies were considered sufficiently rigorous to be included in the review. Each study clearly stated its inclusion criteria, enhancing reproducibility, and described the study population and setting in sufficient detail, facilitating the assessment of generalisability of findings.

The most common potential source of bias was exposure measurement, as HPV vaccination status was self-reported in eight studies, raising concerns about recall bias and exposure misclassification. Only one study verified vaccination status through a national immunisation registry [47]. In contrast, all studies used objective methods for outcome measurement, such as HPV DNA testing through oral samples, which minimizes outcome measurement bias and enhances reliability [50].

**Fig. 2:**
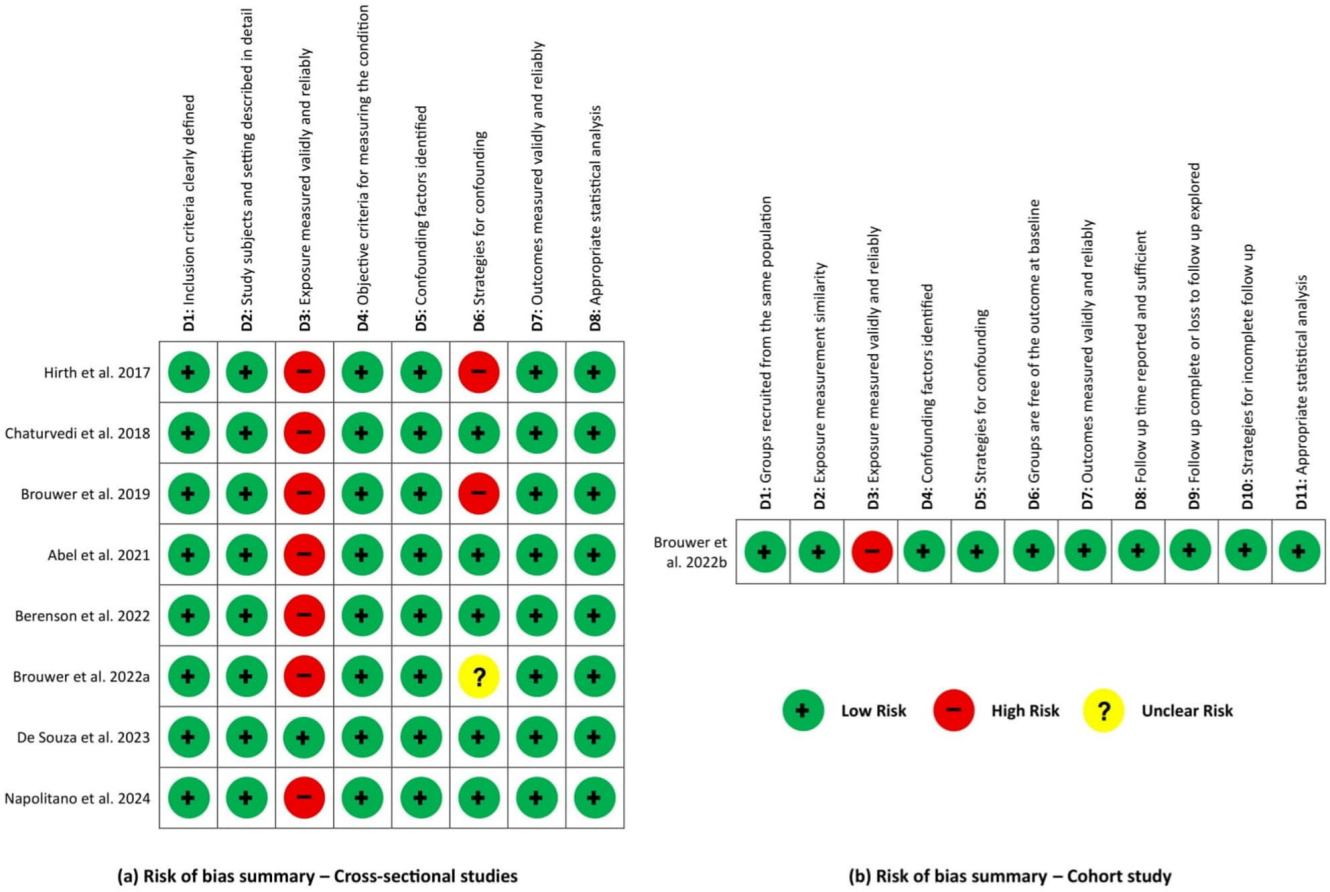
Risk of bias summaries: **a** cross-sectional studies; **b** cohort study

Confounding was appropriately addressed in most studies; however, one study did not adjust for confounding due to low HPV prevalence [40], one conducted descriptive analyses only without statistical control [42], and one relied on univariable regression models [45]. Figure 2 summarises the risk of bias by domain, with detailed assessments provided in Additional file 3. In general, most studies showed low risk in outcome and analysis domains, while exposure measurement remained a consistent concern. Despite these limitations, the overall quality assessment confirms that all included studies are suitable for systematic review inclusion and provide a moderate-to-high level of confidence in the extracted data.

### Oral HPV prevalence and vaccine effectiveness

Across the seven cross-sectional studies [40–44, 47, 48] assessing prevalence of any HPV or vaccine-type HPV infection, vaccinated individuals consistently exhibited lower oral HPV prevalence compared with unvaccinated participants. The two cohort studies [45, 46] found no significant association between vaccination and oral HPV prevalence or incidence. Table 3 summarises these findings across the included studies.

**Table 3:** Oral HPV Prevalence and Incidence by HPV Vaccination Status

Study (Year)	Outcome – Prevalence	Total *n* (%, 95% CI)	Vaccinated *n* (%, 95% CI)	Unvaccinated *n* (%, 95% CI)	*P*-value (Vaccinated vs. Unvaccinated) *	Relative reduction %**
Hirth et al. 2017 [40]	Any HPV	178 (5.6%, 95% CI: 4.4–7.0%)	38 (4.47%, 95% CI: 3.34–6.00%)	140 (5.88%, 95% CI: 4.48–7.68%)	0.18	24%
	VT-HPV (4v)	34	2 (0.16%, 95% CI: 0.04–0.68%)	32 (1.47%, 95% CI: 0.93–2.32%)	**< 0.001**	89.1%
	VT-HPV (9v)	46	6 (0.52%, 95% CI: 0.22–1.21%)	40 (1.80%, 95% CI: 1.15–2.81%)	**0.001**	71.1%
Chaturvedi 2018 [41]	VT-HPV (4v)	33	1 (0.11%, 95% CI: 0.0–0.96%)	32 (1.61%, 95% CI: 1.00–2.47%)	**0.008**	93.2%
Brouwer et al. 2019 [42]	Any HPV	NR	F: (4.0%); M: (8.3%)	F: (4.5%); M: (6.3%)	F: 0.36; M: 0.75	F: 11.1% M: -31.7%
	VT-HPV (4v)	NR	F: (0.1%); M: (0.0%)	F: (1.0%); M: (1.0%)	F: **0.05**; M: **0.02**	F: 90% M: 100%
Abel et al. 2021 [43]	VT-HPV (4v)	62	1-dose: 1 (0.3%, 95% CI: 0.0–0.9%); 2–3 doses: 2 (0.4%, 95% CI: 0.0–1.2%)	59 (1.2%, 95% CI: 0.9–1.6%)	1-dose: **0.01**; 2–3 doses: **0.05**	1-dose: 75% 2–3 doses: 66.7%
Berenson et al. 2022 [44]	Any HPV	687 (6.9%, 95% CI: 6.1–7.9%)	52 (4.22%, 95% CI: 3.23–5.52%)	635 (7.21%, 95% CI: 6.39–8.26%)	**0.001**	41.5%
	VT-HPV (4v)	131 (1.4%, 95% CI: 1.1–1.7%)	6 (0.62%, 95% CI: 0.20–1.85%)	125 (1.46%, 95% CI: 1.18–1.80%)	0.12	57.5%
	VT-HPV (9v)	194 (2.1%, 95% CI: 1.8–2.5%)	12 (1.10%, 95% CI: 0.52–2.28%)	182 (2.23%, 95% CI: 1.87–2.67%)	0.07	50.7%
Brouwer et al. 2022a [45]^†^	Any HPV	34 (10.0%)	16 (10.1%)	15 (9.4%)	NR	-7.4%
	VT-HPV (4v)	21 (6.2%)	(6.3%)	(6.3%)	NR	0%
de Souza et al. 2023 [47]	Any HPV	66 (7.2%)	15 (6.5%, 95% CI: 4.0–10.5%)	51 (7.6%, 95% CI: 5.8–9.9%)	0.583	14.5%
	VT-HPV (4v)	16	1 (0.4%)	15 (2.2%)	**0.042**	81.8%
	VT-HPV (9v)	25	2 (0.9%)	23 (3.4%)	**0.023**	73.5%
Napolitano et al. 2024 [48]	Any HPV	8 (0.7%)	1 (0.2%)	7 (1.5%)	**0.04**	86.7%

Study (Year)	Outcome – Incidence	Total *n* (%, 95% CI)	Vaccinated *n* (%, 95% CI)	Unvaccinated *n* (%, 95% CI)	HR (95% CI) (Vaccinated vs. Unvaccinated) *	Relative reduction %
Brouwer et al. 2022b [46]	Incidence – Any HPV	94 pairs (from a total: 1676)	NR	NR	1.22 (95% CI: 0.87–1.71)	-22%

*F*  females, *M*  males, *NR*  not reported, *HR*  hazard ratio, *OR*  odds ratio, *CI*  confidence interval. Bold values indicate statistically significant differences (*p* ≤ 0.05)

‘Any HPV’ includes any oral HPV genotype, while ‘VT-HPV’ refers to vaccine-type oral HPV

* For prevalence rates, *P*-values refer to statistical tests comparing vaccinated and unvaccinated participants. For incidence, HRs with 95% CIs are presented instead

** Percentages and numbers in the table are reported as in the original studies. Only the values in the final column represent reviewer-calculated synthesis results

^†^ Vaccination status was reported by only 94% of participants; therefore, the sum of vaccinated and unvaccinated cases is less than the total number of HPV-positive cases

#### Vaccine-type HPV (4v)

Seven studies assessed the prevalence of oral infection with HPV genotypes targeted by the quadrivalent vaccine (4v VT-HPV) [40–45, 47]. Six reported lower VT-HPV prevalence among vaccinated participants [40–44, 47], five of which were statistically significant [40–43, 47]. After excluding the study with sex-stratified subgroups [42] and the only study with different study design [45], relative reductions in 4v VT-HPV prevalence ranged from 57.5% to 93.2%, with a mean reduction of 78.5% and a median of 81.8%. Only one study examined the impact of vaccine dose number on oral HPV prevalence, and median and mean values were calculated after averaging dose-specific estimates within that study [43].

They reported lower VT-HPV prevalence in both 1-dose (0.3%, 95% CI: 0.0–0.9%) and 2–3-dose groups (0.4%, 95% CI: 0.0–1.2%) compared to unvaccinated participants (1.2%, 95% CI: 0.9–1.6%). Differences were statistically significant for each vaccinated group relative to the unvaccinated group, but not between vaccinated subgroups, suggesting that partial vaccination may also be associated with reduced VT-HPV prevalence [43]. In the baseline analysis of the cohort study [45], oral HPV prevalence did not significantly differ between groups, with a 4v VT-HPV prevalence of 6.3% in both vaccinated and unvaccinated participants. However, when stratified by age, vaccinated participants in both college-age and older cohorts displayed lower oral HPV prevalence rates. The authors noted that ecological confounding may have obscured vaccine effects when analysing the full cohort [45]. These findings point to a substantial and consistent association between HPV vaccination and lower prevalence of oral VT-HPV, particularly the genotypes (HPV 6, 11, 16, and 18).

#### Any HPV

Seven studies reported the prevalence of oral HPV infection of any genotype by vaccination status [40, 42, 44–48]. While five reported lower prevalence among vaccinated participants [40, 42, 44, 47, 48], only two reached statistical significance [44, 48]. After excluding cohort studies [45, 46] and sex-stratified subgroup analyses [42], relative reductions in prevalence among vaccinated vs. unvaccinated individuals ranged from 14.5% to 86.7%, with a mean reduction of 41.7% and a median of 32.7%. The largest effect was observed in Italy, where prevalence was 0.2% among vaccinated *participants compared with* 1.5% among unvaccinated participants with a *p*-value of 0.04 [48]. More modest reductions were observed in the United States and Australia, where prevalence differences were smaller but consistently favoured vaccination [40, 44, 47]. The cohort studies again diverged from this pattern, with the baseline analysis reporting a slightly higher prevalence among the vaccinated (10.1%) compared to the unvaccinated group (9.4%) for any HPV genotype [45].

In the longitudinal analysis [46], HPV vaccination was associated with a significantly lower incidence of cervicogenital HPV infection, but not with oral HPV incidence. They reported an adjusted hazard ratio of 1.22 (95% CI: 0.87–1.71), indicating no significant reduction in incident oral HPV infection among vaccinated individuals. The authors also observed that oral HPV infections were highly transient, with a mean genotype-specific clearance time of 46 days, which may have limited the ability to detect vaccine-related reductions in oral HPV [46]. Overall, evidence from the cross-sectional studies suggests that HPV vaccination is associated with a reduction in oral infection from any HPV type, though the effect is less consistent and generally smaller in magnitude than the reductions observed for vaccine-type HPV.

#### Vaccine-type HPV (9v)

Three studies assessed oral infection with the nine genotypes targeted by the 9-valent HPV vaccine. All demonstrated lower prevalence among vaccinated individuals, with relative reductions ranging from 50.7% to 73.5%. The mean reduction was 65.1%, with a median of 71.1%. Although these findings indicate reduced oral prevalence of 9v VT-HPV, the observed reductions were smaller than those reported for 4-valent VT-HPV.

### Gender variations in oral HPV prevalence by vaccination status

Of the nine included studies, seven reported oral HPV prevalence separately for male and female participants [40–42, 44–47], four of which further stratified results by both gender and vaccination status [40–42, 44]. Table 4 presents the prevalence of oral HPV infections among males and females by vaccination status, as reported across the included studies.

**Table 4:** Prevalence of vaccine-type (4v) oral HPV infections among males and females by vaccination status

Study	Sex	Total *n* (%, 95% CI)	Vaccinated *n* (%, 95% CI)	Unvaccinated *n* (%, 95% CI)	*P*-value (Vaccinated vs. Unvaccinated)	Relative reduction %*
Hirth et al. 2017 [40]	Males	21 (2.02%, 95% CI: 1.12–3.63)	1 (0.42%, 95% CI: 0.05–3.11)	20 (2.18%, 95% CI: 1.21–3.90%)	NR	80.7%
	Females	13 (0.64%, 95% CI: 0.34–1.20)	1 (0.11%, 95% CI: 0.01–0.82)	12 (0.87%, 95% CI: 0.46–1.65)	NR	87.4%
Chaturvedi et al. 2018 [41]	Males	23	0	23 (2.13%, 95% CI: 1.12–3.65)	**0.007**	100%
	Females	10	1 (0.14%, 95% CI: 0.00–1.21)	9 (0.97%, 95% CI: 0.40–1.95)	0.087	85.6%
Brouwer et al. 2019 [42]	Males	NR	(0.0%)	(1.0%)	**0.02**	100%
	Females	NR	(0.1%)	(1.0%)	**0.05**	90.0%
Berenson et al. 2022 [44]	Males	107 (2.4%, 95% CI: 1.9-3.0%)	5 (2.48%, 95% CI: 0.74–7.94)	102 (2.38%, 95% CI: 1.90–2.97)	0.68	-4.2%
	Females	24 (0.3%, 95% CI: 0.2–0.8%)	1 (0.08%, 95% CI: 0.01–0.58)	23 (0.50%, 95% CI: 0.28–0.90)	**0.04**	84.0%

*NR*  not reported. Bold values indicate statistically significant differences (*p* ≤ 0.05)

* Percentages and numbers in the table are reported as in the original studies. Only the values in the final column represent reviewer-calculated synthesis results

Among the included studies, only four reported the prevalence of oral HPV, specifically quadrivalent vaccine-type oral HPV, stratified by both sex and vaccination status and are presented in Table 4 [40–42, 44]. Three additional studies reported prevalence among males and females but did not further stratify by vaccination status. One of them reported sex-specific prevalence of VT-HPV (4v) [47], while two reported prevalence of any oral HPV genotype [45, 48]. Another study predicted significantly higher probabilities of oral VT-HPV infection in males compared with females [43], but these were modelled estimates rather than observed prevalence data, and the study was therefore excluded from Table 4.

Four studies reported a higher prevalence of oral VT-HPV (4v) in males than females [40, 41, 44, 47]. Notably, the only study including non-binary participants observed prevalence rates of 9.7% in males, 6.5% in females, and 0% in non-binary individuals [47]. In contrast, one study found a higher prevalence in females (10.5%) than in males (9.0%) [45]. An additional study reported only unstratified case counts (two males and six females), without prevalence estimates or vaccination breakdown [48]. Finally, the only study reporting incidence showed a lower incidence of oral HPV in males than females (HR = 0.85, 95% CI: 0.59–1.23), although this difference was not statistically significant [46].

In studies reporting sex-stratified vaccination data [40–42, 44], vaccinated participants consistently exhibited lower oral HPV prevalence than unvaccinated participants. For females, all four studies noted substantial reductions in vaccine-type HPV prevalence, ranging from 84.0% to 90.0% (mean 86.8%, median 86.5%). Among males, the mean relative reduction was 69.1% (median 90.4%). One outlier study [44] reported slightly higher prevalence in vaccinated males (2.48%) compared with unvaccinated males (2.38%), though the difference was not statistically significant (*p* = 0.68). In the other three studies, reductions in males ranged from 80.7% to 100% [40–42], with two studies reporting absence of HPV infection among vaccinated males, corresponding to 100% reduction in prevalence compared to unvaccinated males [41, 42].

Two studies found higher VT-HPV prevalence in vaccinated males compared with vaccinated females [40, 44], while two observed the opposite [41, 42]. Among unvaccinated individuals, three studies consistently reported higher VT-HPV prevalence in males [40, 41, 44], while one reported equal prevalence in both sexes [42].

Overall, these findings suggest potential sex-related differences in oral HPV prevalence. However, limited reporting on variables such as age at vaccination, together with heterogeneity in reporting methods and statistical analyses, restricts direct comparisons and interpretation of gender-specific patterns.

A meta-analysis was not conducted due to substantial heterogeneity across the included studies in terms of outcome definitions, stratification variables, and analytical approaches. The studies differed in the HPV genotypes assessed, with some reporting on any HPV, others focusing on quadrivalent or nonavalent vaccine types, and a few assessing narrower subsets. There was also variation in how results were stratified, including differences by vaccination status, gender, age group, or number of doses received. Furthermore, effect measures were reported inconsistently, with some studies presenting raw prevalence rates, others reporting modelled probabilities or hazard ratios, and some omitting confidence intervals or *p*-values altogether, which complicates statistical synthesis. Importantly, five of the nine studies were based on data from the NHANES surveys in the United States, and several of these analysed overlapping survey cycles, raising the possibility of participant duplication. This potential overlap compromises the assumption of independence required for meta-analysis. Considering these issues, a narrative synthesis was conducted, incorporating relative reductions, ranges, means, and medians where applicable.

## Discussion

### Principal findings

This systematic review is the first to specifically evaluate the effectiveness of gender-neutral HPV vaccination programmes in reducing HPV-associated OCs, based exclusively on studies involving gender-neutral populations. By examining the prevalence of detectable oral HPV, it offers timely evidence that may help understand how vaccination could influence HPV-associated cancer risk.

Nine observational studies published between 2017 and 2024 were included. The focus on observational studies, particularly cross-sectional designs, aligns with the objective of gathering real-world data on oral HPV prevalence and vaccination impact. It is also important to acknowledge that establishing causality in observational studies presents challenges [51]. While the evidence suggests reduced oral HPV prevalence following vaccination, particularly for vaccine-type strains, causal claims should be interpreted with caution. Further prospective longitudinal studies and clinical trials are warranted to strengthen the evidence base.

Most included studies reported a clear trend toward reduced oral HPV prevalence among vaccinated individuals. This trend was observed across both males and females, although the magnitude of reduction varied between studies. Notably, two studies reported a 100% reduction in VT-HPV prevalence among vaccinated males compared with unvaccinated males [41, 42], highlighting the importance of increasing male inclusion in HPV immunisation programmes. Evidence among females also demonstrated reduced prevalence following vaccination, supporting the potential effectiveness of HPV vaccination across sexes.

### Comparison with existing literature

This aligns with the findings of another systematic review, which also advocates for gender-neutral vaccination policies as an effective strategy to reduce oral HPV prevalence, particularly among males [29]. They also highlight the limitations of female-only programmes in protecting at-risk male populations, such as MSM and unvaccinated subgroups. In addition, a scoping review similarly reported a 72–93% reduction in oral VT-HPV prevalence among vaccinated individuals, noted variation in effect by gender, and emphasised the need for further longitudinal studies [52].

While most cross-sectional studies reported lower oral HPV prevalence among vaccinated individuals, the cohort study did not demonstrate a statistically significant difference in oral HPV incidence between vaccinated and unvaccinated participants. This divergence likely reflects both biological and methodological factors. Oral HPV infection is typically transient, characterised by rapid natural clearance, which reduces the probability of detecting incident infections during scheduled longitudinal follow-up [46]. By contrast, cross-sectional designs capture infections present at a single time point, including transient episodes, thereby increasing the likelihood of detecting differences according to vaccination status [49]. Additional methodological factors, including self-reported vaccination status, residual confounding, and variable sampling intervals, may also have contributed to these differences.

Overall, the sex pattern observed across this review, where several included studies reported higher oral HPV prevalence in males than females, appears broadly consistent with a nationally representative study from the U.S., which found higher prevalence in men than women for both any oral HPV (11.5% vs. 3.3%) and high-risk oral HPV (6.8% vs. 1.2%) [18]. However, direct comparison of absolute prevalence estimates is limited because most included studies reported oral VT-HPV (primarily 4v types) and frequently stratified outcomes by vaccination status, whereas that study reflects population-level prevalence across a broader set of HPV types.

In this context, the higher burden of HPV-associated oral and oropharyngeal cancers among males reinforces the relevance of gender-neutral HPV vaccination strategies targeting high-risk HPV types, particularly HPV 16 and 18, which are covered by current vaccines [19, 29].

### Implications for research and policy

The findings of this review highlight the importance of expanding and promoting gender-neutral HPV vaccination programmes globally. Such initiatives help reduce health inequalities by ensuring that both males and females have equal access to the protective benefits of HPV vaccines [53]. This approach not only promotes equity but also maximises public health impact, particularly given the evidence that men are at increased risk of acquiring HPV-associated OCs compared to women [26]. Moreover, gender-neutral vaccination could address additional health inequalities, such as among MSM who may not benefit from herd immunity through female vaccination [54]. These programmes have the potential to help reduce the burden of HPV-associated oral and oropharyngeal cancers, ultimately saving lives and alleviating the burden on healthcare systems. Policymakers and healthcare authorities should consider these findings as they shape vaccination policies and work towards a future where gender-neutral HPV vaccination programmes are universally accessible and actively promoted.

### Strengths and limitations

A key strength of this review is the inclusion of recent studies, reflecting contemporary vaccination trends following the adoption of gender-neutral HPV vaccination programmes in several countries. The review also captured a broad age distribution, with nearly half the studies focusing on younger adults (18–36 years) and the remainder including older age groups (reaching 70 years). However, these refer to participants’ ages at the time of sampling, not at the time of vaccination. Therefore, the observed prevalence patterns in older age groups do not necessarily reflect vaccine-induced protection, as HPV infection status at the time of vaccination is unknown, and are more likely to reflect natural, age-related differences in oral HPV exposure and clearance. This review identified gender-specific trends in oral HPV prevalence, underscoring the importance of sex-stratified analyses when evaluating population-level vaccination outcomes. While only one study in our review stratified results by number of doses [43], these findings provide a preliminary view into the potential population-level impact of single-dose vaccination. This is particularly relevant given the increasing global momentum toward single-dose schedules, especially in settings with limited resources [20]. However, further research is needed to directly compare oral HPV prevalence outcomes across dose regimens and to confirm the long-term effectiveness of reduced-dose strategies in diverse populations. PRISMA guidelines were followed in this review and a pre-defined criteria was applied, ensuring methodological transparency and reproducibility. The review employed a rigorous and sensitive search strategy using multiple reputable databases (e.g. MEDLINE, Embase via Ovid), as well as, grey literature and trial registries, increasing sensitivity. In addition, the use of the JBI Critical Appraisal Checklists to assess risk of bias enhances the reliability of the findings and enables readers to interpret the results with confidence.

This review has several limitations that should be considered when interpreting the findings. First, the limited number of included studies (*n* = 9) and the predominance of U.S.-based data may limit the generalisability of findings despite the overall consistency in trends. The review included only studies published in English, which may have introduced language bias. By exclusively including studies with gender-neutral samples, the findings are highly relevant to countries where such programmes are implemented but may not be generalisable to low- and middle-income countries where male vaccination remains costly or not yet implemented. In addition, studies focusing entirely on high-risk populations (e.g., HIV patients, MSM, or sex workers) were excluded to enhance applicability to general populations targeted by national vaccination efforts. However, this limits insight into vaccine effectiveness in vulnerable populations with elevated risk of oral HPV infection.

All included studies were observational, limiting causal inference as mentioned earlier. In addition, because most included studies used cross-sectional designs and assessed vaccination status and oral HPV infection at a single time point, baseline oral HPV status prior to vaccination was generally unavailable, which limits interpretation of whether participants were HPV-negative at the time of vaccination. Although most studies adjusted for variables such as age and demographics, two lacked comprehensive adjustment, and one used only univariable regression, decreasing internal validity. Additionally, eight of the nine studies relied solely on self-reported vaccination status, introducing potential recall bias, while only one study verified vaccination using immunisation records. A major limitation is the possibility of sample overlap because five studies have selected their samples from the NHANES datasets in the U.S. using overlapping cycles (years). While NHANES is nationally representative and enhances generalisability, the overlap may have resulted in the inclusion of the same individuals multiple times. This overlap, combined with heterogeneity in outcome definitions, stratification of results, and analytical approaches posed significant challenges to quantitative synthesis. Therefore, the decision not to conduct a meta-analysis was based on these methodological and data-related considerations, as any pooled estimates would likely have been misleading. Moreover, most included studies reported participant age at outcome collection but not at vaccination. Since HPV vaccines are most effective when administered before exposure [21], this missing information limits the ability to interpret findings in relation to timing of vaccine delivery. Lastly, none of the included studies accounted for the possibility of assortative mating based on vaccination status. For instance, vaccinated individuals may be more likely to choose partners who are also vaccinated, thereby reducing exposure to VT-HPV strains aside from the vaccine’s protective efficacy. A previous study suggested that such behavioural patterns exist among females [55].

It is important to note that the detection of HPV DNA reflects point prevalence and does not necessarily indicate active or persistent infection. Accordingly, the prevalence data presented in this review should be interpreted with caution and not directly equated with infection.

### Future research

Our systematic review identifies several areas for future research. First, the study outcomes warrant further investigation to understand the underlying factors influencing vaccine effectiveness. Prospective studies could shed light on the long-term impact of HPV vaccination on oral HPV prevalence. Additionally, research examining the impact of different vaccine dosing regimens, including single-dose strategies as demonstrated by one of the included studies, could help refine current vaccination guidelines, especially in low-resource settings. Finally, more data are needed from diverse populations and geographic regions, including low- and middle-income countries where HPV vaccination coverage remains low and prevalence of oral HPV is underreported [56, 57].

## Conclusion

This systematic review provides evidence of an association between HPV vaccination and lower prevalence of detectable oral vaccine-type HPV DNA among males and females. Including males in national HPV immunisation strategies is crucial for promoting equity and maximising public health impact, especially in light of the disproportionate burden of HPV-associated OPCs among men. Sex disparities in oral HPV prevalence were observed with and without vaccination. These findings support the potential effectiveness of gender-neutral HPV vaccination programmes on reducing the burden of HPV-associated OCs. However, further prospective studies are essential to strengthen causal inference and guide evidence-based vaccination policies.

## Supplementary Information

Additional file 1: Full search strategies. Complete search strategies for each database (Medline, Embase) and grey literature.

Additional file 2: Screening by full-text results with reasons of exclusion. Table of studies excluded at the full-text screening stage, with reasons based on the review’s eligibility criteria.

Additional file 3: JBI Critical Appraisal Checklists. Full risk of bias assessments for each included study using the JBI Critical Appraisal Checklists.

Additional file 4: PRISMA 2020 checklist. Completed PRISMA 2020 checklist outlining adherence to systematic review reporting standards.

## Data Availability

The datasets supporting the conclusions of this article are included within the article and its additional files.

## Abbreviations

ASIR: Age-standardised incidence rate
CI: Confidence interval
EUCTR: European Union Clinical Trials Register
FDA: Food and Drug Administration
FDI: World Dental Federation
HPV: Human papillomavirus
HR: Hazard ratio
JBI: Joanna Briggs Institute
MHOC: Michigan HPV and Oropharyngeal Cancer
MSM: Men who have sex with men
NHANES: National Health and Nutrition Examination Survey
NR: Not reported
NA: Not applicable
OCs: Oral cancers
OPCs: Oropharyngeal cancers
OR: Odds ratio
PICOS: Patient, Intervention, Comparison, Outcome, and Study design
PRISMA: Preferred Reporting Items for Systematic Reviews and Meta-Analyses
U.S.: United States
VT-HPV: Vaccine-type HPV
WHO: World Health Organization
WHO ICTRP: World Health Organization’s International Clinical Trials Registry Platform
