# Supplementary material for: The effectiveness of gender-neutral HPV vaccination programmes in preventing HPV-associated oral cancers: a systematic review
Source: BMC Cancer. 2026 Apr 16;26:678. doi: 10.1186/s12885-026-15979-3 (PMC13214456; doi:10.1186/s12885-026-15979-3)
Supplement: Supplementary file 1 — Additional file 1: Full search strategies. Complete search strategies for each database (Medline, Embase) and grey literature. [file 12885_2026_15979_MOESM1_ESM.pdf]

Database: Embase <1974 to 2025 February 07>

Search Strategy:

1 exp Wart virus/ (47414)

2 (human adj2 papilloma*).ab,kf,ti. (69144)

3 (hpv or papillomavir*).ab,kf,ti. (93372)

4 1 or 2 or 3 (103959)

5 *vaccine/ (35546)

6 "vaccin*".ab,kf,ti. (534774)

7 *vaccination/ (92579)

8 5 or 6 or 7 (543950)

9 4 and 8 (25474)

10 exp Human papilloma virus vaccine/ (19957)

11 (hpv adj2 vaccin*).ab,kf,ti. (16823)

12 (2vHPV or 4vHPV or 9vHPV).ab,kf,ti. (306)

13 (bivalent adj2 hpv).ab,kf,ti. (335)

14 (quadrivalent adj2 hpv).ab,kf,ti. (965)

15 (9-valent adj2 hpv).ab,kf,ti. (259)

16 (Cervarix* or gardasil*).ab,kf,ti. (1166)

17 9 or 10 or 11 or 12 or 13 or 14 or 15 or 16 (30514)

18 mouth disease/ (26472)

19 exp mouth/ (267109)

20 exp oropharynx/ (26273)

21 (oral or oropharyngeal).ab,kf,ti. (1089579)

22 18 or 19 or 20 or 21 (1298761)

23 exp papillomavirus infection/ (50211)

24 22 and 23 (5652)

25 exp neoplasm/ (6151185)

26 22 and 25 (269179)

27 exp mouth tumor/ (132950)

28 exp oropharynx tumor/ (30086)

29 exp mouth squamous cell carcinoma/ (16830)

30 exp oropharynx squamous cell carcinoma/ (4165)

31 (oral adj2 cancer*).ab,kf,ti. (29252)

32 (oral adj2 neoplasm*).ab,kf,ti. (562)

33 (oral adj2 malignan*).ab,kf,ti. (4393)

34 (oropharyn* adj2 cancer*).ab,kf,ti. (9056)

35 (oropharyn* adj2 neoplasm*).ab,kf,ti. (366)

36 (oropharyn* adj2 malignan*).ab,kf,ti. (281)

37 24 or 26 or 27 or 28 or 29 or 30 or 31 or 32 or 33 or 34 or 35 or 36 (334296)

38 exp epidemiology/ (4962011)

39 incidence/ (647691)

40 prevalence/ (1062898)

41 morbidity/ (436914)

42 38 or 39 or 40 or 41 (4962011)

43 17 and 37 and 42 (1135)

44 limit 43 to yr="2009 -Current" (1076)

Database: Ovid MEDLINE(R) and Epub Ahead of Print, In-Process, In-Data-Review & Other Non-Indexed Citations, Daily and Versions <1946 to February 07, 2025>

Search Strategy:

1 exp Human Papillomavirus Viruses/ (8608)
2 (human adj2 papilloma*).ab,kf,ti. (56549)
3 (hpv or papillomavir*).ab,kf,ti. (70452)
4 1 or 2 or 3 (73154)
5 "vaccin*".ab,kf,ti. (462931)
6 4 and 5 (19056)
7 exp Papillomavirus Vaccines/ (11167)
8 (hpv adj2 vaccin*).ab,kf,ti. (12804)
9 (2vHPV or 4vHPV or 9vHPV).ab,kf,ti. (245)
10 (bivalent adj2 hpv).ab,kf,ti. (265)
11 (quadrivalent adj2 hpv).ab,kf,ti. (666)
12 (9-valent adj2 hpv).ab,kf,ti. (205)
13 (Cervarix* or gardasil*).ab,kf,ti. (796)
14 6 or 7 or 8 or 9 or 10 or 11 or 12 or 13 (20090)
15 Diagnosis, Oral/ (2019)
16 exp Mouth/ (330839)
17 exp Oropharynx/ (15733)
18 (oral or oropharyngeal).ab,kf,ti. (793230)
19 15 or 16 or 17 or 18 (1080889)
20 Papillomavirus Infections/ (36572)
21 19 and 20 (5177)
22 Neoplasms/ (536607)
23 19 and 22 (13512)
24 exp Mouth Neoplasms/ (80354)
25 exp Oropharyngeal Neoplasms/ (10633)
26 (oral adj2 cancer*).ab,kf,ti. (23381)
27 (oral adj2 neoplasm*).ab,kf,ti. (427)
28 (oral adj2 malignan*).ab,kf,ti. (3871)

29 (oropharyn* adj2 cancer*).ab,kf,ti. (6177)

30 (oropharyn* adj2 neoplasm*).ab,kf,ti. (265)

31 (oropharyn* adj2 malignan*).ab,kf,ti. (167)

32 "Squamous Cell Carcinoma of Head and Neck"/ (13798)

33 21 or 23 or 24 or 25 or 26 or 27 or 28 or 29 or 30 or 31 or 32 (121528)

34 exp Morbidity/ (687196)

35 (incidence or prevalence).ab,kf,ti. (1800994)

36 34 or 35 (2012187)

37 14 and 33 and 36 (614)

38 limit 37 to yr="2009 -Current" (576)

Grey literature and trials registries

- ***ClinicalTrials.gov***

Search Strategy:
Condition or disease: Human Papilloma Virus Infection
Other terms: Oral Cancer
Intervention/Treatment: HPV Vaccine
Location: Not specified
Study Status: All studies (Selected)
    Results: 33 Trials

- ***World Health Organization's International Clinical Trials Registry Platform (WHO ICTRP)***

Search strategy: In the Advanced Search page:
Human Papillomavirus Infection **in the Condition**
HPV Vaccine **in the Intervention**
Recruitment status is ALL
    Results: 24 Trials

- ***European Union Clinical Trials Register***

Search strategy: Oral Human Papillomavirus Infection **AND** HPV Vaccine
    Results: 24 Trials
