# Supplementary material for: The effectiveness of gender-neutral HPV vaccination programmes in preventing HPV-associated oral cancers: a systematic review
Source: BMC Cancer. 2026 Apr 16;26:678. doi: 10.1186/s12885-026-15979-3 (PMC13214456; doi:10.1186/s12885-026-15979-3)
Supplement: Supplementary file 3 — Additional file 3: JBI Critical Appraisal Checklists. Full risk of bias assessments for each included study using the JBI Critical Appraisal Checklists. [file 12885_2026_15979_MOESM3_ESM.pdf]

JBI CRITICAL APPRAISAL CHECKLIST FOR ANALYTICAL CROSS SECTIONAL STUDIES

Reviewer________________________________Date__________________________

Author_________________________________Year________ Record Number________

	Yes	No	Unclear	Not applicable
1. Were the criteria for inclusion in the sample clearly defined?	☐	☐	☐	☐
2. Were the study subjects and the setting described in detail?	☐	☐	☐	☐
3. Was the exposure measured in a valid and reliable way?	☐	☐	☐	☐
4. Were objective, standard criteria used for measurement of the condition?	☐	☐	☐	☐
5. Were confounding factors identified?	☐	☐	☐	☐
6. Were strategies to deal with confounding factors stated?	☐	☐	☐	☐
7. Were the outcomes measured in a valid and reliable way?	☐	☐	☐	☐
8. Was appropriate statistical analysis used?	☐	☐	☐	☐

Overall appraisal: Include ☐ Exclude ☐ Seek further info ☐

Comments (Including reason for exclusion)

JBI CRITICAL APPRAISAL CHECKLIST FOR COHORT STUDIES

Reviewer________________________________Date__________________________

Author_________________________________Year________ Record Number________

	Yes	No	Unclear	Not applicable
1. Were the two groups similar and recruited from the same population?	☐	☐	☐	☐
2. Were the exposures measured similarly to assign people to both exposed and unexposed groups?	☐	☐	☐	☐
3. Was the exposure measured in a valid and reliable way?	☐	☐	☐	☐
4. Were confounding factors identified?	☐	☐	☐	☐
5. Were strategies to deal with confounding factors stated?	☐	☐	☐	☐
6. Were the groups/participants free of the outcome at the start of the study (or at the moment of exposure)?	☐	☐	☐	☐
7. Were the outcomes measured in a valid and reliable way?	☐	☐	☐	☐
8. Was the follow up time reported and sufficient to be long enough for outcomes to occur?	☐	☐	☐	☐
9. Was follow up complete, and if not, were the reasons to loss to follow up described and explored?	☐	☐	☐	☐
10. Were strategies to address incomplete follow up utilized?	☐	☐	☐	☐
11. Was appropriate statistical analysis used?	☐	☐	☐	☐

Overall appraisal: Include ☐ Exclude ☐ Seek further info ☐

Comments (Including reason for exclusion)

Quality Assessment of the included studies based on JBI Critical Appraisal checklist for analytical cross sectional studies

Question Number	Hirth et al. 2017	Chaturvedi et al. 2018	Brouwer et al. 2019	Abel et al. 2021	Berenson et al. 2022	Brouwer et al. 2022a	De Souza et al. 2023	Napolitano et al. 2024
**1**	Yes	Yes	Yes	Yes	Yes	Yes	Yes	Yes
**2**	Yes	Yes	Yes	Yes	Yes	Yes	Yes	Yes
**3**	No (Vaccination status was self-reported without verification from national records)	No (Vaccination status was self-reported without verification from national records)	No (Vaccination status was self-reported without verification from national records)	No (Vaccination status was self-reported without verification from national records)	No (Vaccination status was self-reported without verification from national records)	No (Vaccination status was self-reported without verification from national records)	Yes (Vaccination status was confirmed via the Australian Immunisation Register (AIR))	No (Vaccination status was self-reported without verification from national records)
**4**	Yes	Yes	Yes	Yes	Yes	Yes	Yes	Yes
**5**	Yes	Yes	Yes	Yes	Yes	Yes	Yes	Yes
**6**	No (Authors noted inability to adjust due to low HPV prevalence)	Yes	No (Comparisons by vaccination status were descriptive; confounding was not statistically controlled)	Yes	Yes	Unclear (Only univariable regression models were used)	Yes	Yes
**7**	Yes	Yes	Yes	Yes	Yes	Yes	Yes	Yes
**8**	Yes	Yes	Yes	Yes	Yes	Yes	Yes	Yes
**Overall**	Include	Include	Include	Include	Include	Include	Include	Include

Quality Assessment of the included studies based on JBI Critical Appraisal checklist for cohort studies

Question Number	Brouwer et al. 2022b
**1**	Yes
**2**	Yes
**3**	No<br>(Vaccination status was self-reported without verification from national records)
**4**	Yes
**5**	Yes
**6**	Yes
**7**	Yes
**8**	Yes
**9**	Yes
**10**	Yes
**11**	Yes
**Overall**	Include

Study	Study Design	Critical Appraisal Tool	Score	Overall Appraisal
**Hirth et al. 2017**	Cross-sectional	JBI Critical Appraisal Checklist for Analytical Cross-sectional Studies	6/8	Include
**Chaturvedi et al. 2018**			7/8	Include
**Brouwer et al. 2019**			6/8	Include
**Abel et al. 2021**			7/8	Include
**Berenson et al. 2022**			7/8	Include
**Brouwer et al. 2022a**			6/8	Include
**De Souza et al. 2023**			8/8	Include
**Napolitano et al. 2024**			7/8	Include
**Brouwer et al. 2022b**	Cohort	JBI Critical Appraisal Checklist for Cohort Studies	10/11	Include
